# GnRHa/Stanozolol Combined Therapy Maintains Normal Bone Growth in Central Precocious Puberty

**DOI:** 10.3389/fendo.2021.678797

**Published:** 2021-06-09

**Authors:** Shunye Zhu, Lingli Long, Yue Hu, Ying Tuo, Yubin Li, Zhenhua Yu

**Affiliations:** ^1^ Department of Pediatrics, The Third Affiliated Hospital, Sun Yat-sen University, Guangzhou, China; ^2^ Research Center of Translational Medicine, The First Affiliated Hospital, Sun Yat-sen University, Guangzhou, China; ^3^ Department of Pathology, The First Affiliated Hospital, Sun Yat-sen University, Guangzhou, China; ^4^ Reproductive Medicine Center, The First Affiliated Hospital, Sun Yat-sen University, Guangzhou, China; ^5^ Department of Neurosurgery, The First Affiliated Hospital, Sun Yat-sen University, Guangzhou, China

**Keywords:** CPP, GnRHa, ST, bone growth, chondrogenic differentiation

## Abstract

**Background:**

Gonadotropin-releasing hormone agonist (GnRHa) is the gold standard in the treatment of Central Precocious Puberty (CPP) with progressive puberty and accelerative growth. However, GnRHa treatment is reported to result in growth deceleration and prevents growth plate development which leads to a reduction in height velocity. Stanozolol (ST) has been used to stimulate growth in patients with delayed growth and puberty, nevertheless, the effects and mechanisms of ST on CPP with GnRHa treatment are currently unclear.

**Methods and Results:**

In the current study, we recorded the following vital observations that provided insights into ST induced chondrogenic differentiation and the maintenance of normal growth plate development: (1) ST efficiently prevented growth deceleration and maintained normal growth plate development in rats undergoing GnRHa treatment; (2) ST suppressed the inhibitory effect of GnRHa to promote chondrogenic differentiation; (3) ST induced chondrogenic differentiation through the activation of the JNK/c-Jun/Sox9 signaling pathway; (4) ST promoted chondrogenic differentiation and growth plate development through the JNK/Sox9 signaling pathway *in vivo*.

**Conclusions:**

ST mitigated the inhibitory effects of GnRHa and promoted growth plate development in rats. ST induced the differentiation of chondrocytes and maintained normal growth plate development through the activation of JNK/c-Jun/Sox9 signaling. These novel findings indicated that ST could be a potential agent for maintaining normal bone growth in cases of CPP undergoing GnRHa treatment.

## Introduction

Central Precocious Puberty (CPP) refers to premature activation of the hypothalamic-pituitary-gonadal (HPG) axis, resulting in the early development of secondary sexual characteristics (1, 2). The classical definition of precocious puberty is the development of secondary sexual characteristics before the age of 8 years or menarche before the age of 9 years in girls and any secondary sexual characteristic before the age of 9 years in boys (1). Most cases of CPP are idiopathic and seen in girls. In contrast, most boys with CPP have an identifiable cause (2). The treatment of precocious puberty aims to: interrupt sexual maturation until the normal age for pubertal development is reached; revert or stabilize sexual characteristics; delay skeletal maturation; and preserve normal height potential (3–5).

Gonadotropin-releasing hormone agonist (GnRHa) is the standard agent for the treatment of CPP with progressive puberty and accelerative growth (6–8). The efficacy and safety of GnRHa treatment for CPP have been well described (1); however, recent studies demonstrate that GnRHa treatment causes growth deceleration and prevents growth plate development which leads to a marked reduction in height velocity (9–11). Thus, an agent that can mitigate the effects of GnRHa and promote bone growth and maturation is necessary in the treatment of CPP. Previous studies suggest that when height velocity is reduced (<4 cm/year), growth hormone (GH) should be added to the GnRHa treatment (1, 12). A few studies have assessed the effect of GH administration on the height of patients with CPP, and some show a positive effect of GH/GnRHa therapy in children with decreased growth during GnRHa therapy (13, 14). However, there are some disadvantages of GH treatment: its cost is high and its application is inconvenient (15). Other treatments such as estrogen mini-dose replacement, to overcome the decreased growth plate development that is induced by GnRHa, have been reported (16). Previous studies show that mini-dose estrogen treatment can normalize the slowdown of growth rate during GnRHa therapy in patients with CPP (10). However, estrogen has the potential effect of accelerating bone maturation, and dosage is difficult to control because of individual differences (16).

Stanozolol (ST), a non-aromatizable androgen that has a high anabolic to androgenic ratio has been used to stimulate growth and final adult height of patients with other disease such as Turner syndrome (TS) (17). ST also used to treat some serious disorders like aplastic anemia and hereditary angioedema. It is also indicated as an adjunct therapy for the treatment of vascular disorders and growth failures (18). The efficacy and safety of ST treatment have been well described. Patients with TS who were treated with oxandrolone, height velocity was increased without bone age progression (19). Moreover, our previous study shows that ST promotes proliferation of growth plate chondrocytes (15). Together, these studies indicate that anabolic steroid hormones may promote long bone growth without increasing bone age to achieve the final adult height. However, the mechanism of action of ST on growth plates in patients with CPP is still unknown.

The purpose of the current study was to verify the chondro-inductive capacity of ST in rats undergoing GnRHa treatment and to explore the underlying mechanism, which may provide useful information for its potential clinical application in CPP.

## Materials and Methods

### Experimental Animals

All animal experiments were approved by the Medical Ethics Committee of the First Affiliated Hospital of Sun Yat-sen University and carried out in accordance with the U.K. Animals (Scientific Procedures) Act, 1986 and associated guidelines. Normal healthy Sprague-Dawley rats were provided by the experimental animal center of Sun Yat-sen University and housed in a standard animal room with food and water ad libitum under controlled conditions of humidity (50%-70%) and temperature (21°C ± 2°C) and under a 12 h light/12 h dark lighting schedule.

To evaluate the effect of GnRHa and ST, 2.5mg/kg of GnRHa (Diphereline, Ipsen Pharma Biotech, France) was injected intramuscularly once every 2 weeks and 10 mg/kg of ST (Sigma-Aldrich, MO, USA) was injected once a day. PBS as a negative control. Rats were euthanized at 7 weeks of age. Hind limb specimens were dissected and fixed in 4% paraformaldehyde for histological analyses (n = 5 animals per group) (15).

### Histological Analyses

All specimens were obtained from rats post-mortem and fixed with 4% paraformaldehyde. For histologic analysis, specimens were decalcified in 0.5 M EDTA (Sigma-Aldrich, MO, USA) at 4°C. Paraffin-embedded sections were stained with hematoxylin and eosin (H&E) and Safraini O Fast Green (SOFG) to evaluate general structures and growth plate development. Immunohistochemical analyses of the specimens were conducted using specific antibodies. Tissue sections were quantitated according to the number of positive cells per unit area as previously described (20).

### Immunofluorescence

Tissue sections were fixed in 4% PFA for 30 min and permeabilized with 0.3% Triton X-100 for 30 min. Blocking was performed with 5% normal goat serum for 1 h. The tissue sections and the cells were incubated overnight at 4°C in primary antibodies against the following antigens: anti-Sox9 antibody (Abcam, UK; 1:200) and anti-p-JNK antibody (Abcam; 1:200). After washing three times in PBS, the primary antibodies were probed with the secondary antibodies Alexa Fluor 594 goat anti-rabbit (1:500, Invitrogen, Carlsbad, CA, USA) and Alexa Fluor 594 goat anti-mouse (1:500, Invitrogen; Carlsbad, CA, USA) for 1 h at room temperature. Finally, the coverslips were washed in PBS three times and mounted using Prolong Gold Antifade Reagent containing 4′-6-diamidino-2-phenylindole (DAPI) (Molecular Probes, Invitrogen). The targeted marker-positive cells in each visual field were counted under a fluorescence microscope (Carl Zeiss Axio Observer Z1, Zeiss, Oberkochen, Germany).

### Cell Preparation and Treatment

The pre-chondrocyte cell line ADTC5 cells (ATCC, Manassas, VA, USA), from which we acquired the chondrocyte phenotype in the post confluent culture, were maintained in DMEM/F12 (Invitrogen) supplemented with 10% fetal bovine serum (FBS; Invitrogen, Carlsbad, CA, USA) and 1% penicillin/streptomycin in a humidified incubator at 37°C and 5% CO_2_. When confluent, the cells were detached using a trypsin (0.25%)-EDTA (1 mM) (Sigma-Aldrich, MO, USA) solution and subcultured in 6-well or 12-well plates (21).

Human bone mesenchymal stem cells (hBMSC) were immediately isolated and purified from bone marrow samples using density gradient centrifugation as previously described (22). Briefly, hBMSC were isolated from bone marrow blood collected during surgeries. The blood was suspended in low glucose DMEM (Invitrogen, Carlsbad, CA, USA) supplemented with 10% FBS (Gibco, Australia) and 1% penicillin/streptomycinin in a humidified incubator at 37°C and 5% CO_2_. When confluent, the cells were detached using a trypsin (0.25%)-EDTA (1 mM) (Sigma-Aldrich, MO, USA) solution and subcultured in 6-wells or 12-wells plates.

To assess chondrogenic differentiation, 1×10^5^ ATDC5 cells or hBMSC were resuspended in control medium and seeded as micromasses in 24-well plates. ATDC5 cells were allowed to attach for 1 h at 37°C, then 0.5 ml of chondrogenic medium, which contained 10 ng/ml recombinant rat TGFβ (R&D) and 50 μM l-ascorbic acid 2-sulfate (Sigma-Aldrich, MO, USA), was added to the wells. Medium was changed every day and after 9 d, micromasses were either stained with alcian blue or the cells were used for PCR and WB experiments (21).

To investigate the effect of GnRHa and Stanozolol on chondrogenic differentiation, 5nM GnRHa and 10nM Stanozolol were added into ATDC5 cells.

### Quantitative Real-Time PCR

For analysis of gene expression, total RNA was extracted from cells according to the manufacturer’s protocol, and 2 µg of total DNA-free RNA was used to synthesize cDNA using a ReverTra Ace qPCR RT Kit (Toyobo, Osaka, Japan). The reactions were set up in 96-well plates using 1 µl cDNA with Thunderbird SYBR qPCR Mix (Toyobo, Osaka, Japan), to which gene-specific forward and reverse PCR primers were added. QRT-PCR was performed under the following conditions: 95°C for 10 min, followed by 40 cycles of 95°C for 10 s and 55°C for 34 s. These analyses were performed to detect COL-X, COL-II, MMP13, Aggrecan, and Sox9 expression, and β-actin was used as an internal control. Primer sequences are listed in **Tables 1** and **2**.

**Table 1:** Primers for RT-qPCR analysis of gene expression for rats.

Primer	5’ Forward 3’	5’ Reverse 3’
Actin	CGTGCGTGACATCAAAGAGAAG	CGTTGCCAATAGTGATGACCTG
COL-X	GTTCTTGACCCTGGTTCA	CTGAGGGACCTGGGTGT
COL-II	GGGAATGTCCTCTGCGATGAC	GAAGGGGATCTCGGGGTTG
MMP13	TCCCTGGAATTGGCAACAAAG	GCATGACTCTCACAATGCGATTAC
Aggrecan	TTCCACCAGTGCGATGCAG	TGGTGTCCCGGATTCCGTA
Sox9	GAGCCGGATCTGAAGAGGGA	GCTTGACGTGTGGCTTGTTC

**Table 2:** Primers for RT-qPCR analysis of gene expression for humans.

Primer	5’ Forward 3’	5’ Reverse 3’
Actin	TATTGGCAACGAGCGGTTC	ATGCCACAGGATTCCATACCC
COL-X	ATGCTGCCACAAATACCCTTT	GGTAGTGGGCCTTTTATGCCT
COL-II	TGGACGATCAGGCGAAACC	GCTGCGGATGCTCTCAATCT
MMP13	ACTGAGAGGCTCCGAGAAATG	GAACCCCGCATCTTGGCTT
Aggrecan	CACTGTTACCGCCACTTCCC	GACATCGTTCCACTCGCCCT

### Western Blot

Cells were lysed in RIPA buffer, total protein was extracted, and the protein concentration was determined with a BCA assay. A 10% SDS-PAGE gel was loaded with 20 µg of total protein, and the separated proteins were transferred by electro blotting to PVDF membranes. The membranes were blocked with 5% non-fat dry milk in TBST (50 mM Tris, pH 7.6, 150 mM NaCl, 0.1% Tween 20) and incubated with the primary antibody overnight at 4°C in 5% non-fat dry milk in TBST. Immunolabeling was detected using ECL reagent (Invitrogen, Carlsbad, CA, USA). The antibodies used for western blot were from the following sources: anti-Sox9 antibody (Abcam, UK; 1:1000), anti-COL-X antibody (Abcam, UK; 1:1000), anti-COL-II antibody (Abcam, UK; 1:1000), anti-MMP13 antibody (Abcam, UK; 1:500), anti-GAPDH antibody (Sigma-Aldrich, MO, USA; 1:10000), and anti-β-Actin antibody (Sigma-Aldrich, MO, USA; 1:10000).

### RNA Interference

The small interfering RNA (siRNA) duplexes were constructed by GenePharma (GenePharma Co., Suzhou, China). The siRNA duplexes are listed in **Table 3**. Cells were plated at a concentration of 1 x 10^5^ cells/well in 6-well plates and transduced with the small interfering RNA (siRNA) using lipofectamine RNAiMAX transfection reagent (Invitrogen, Carlsbad, CA, USA) according to the manufacturer’s instructions. Different amounts of 20 μM siRNA duplexes were mixed with 5 μl/well of transfection reagent and Opti-MEM reduced-serum medium (Invitrogen, Carlsbad, CA, USA) to a total volume of 500 μl and incubated for 20 min. The mixture was applied to cells for 16 h at 37°C in 5% CO_2_.

**Table 3:** siRNA sequences for RNA interference.

Gene	Sense	Antisense
Control	UAACGACGCGACGACGUAATT	UUACGUCGUCGCGUCGUUATT
c-Jun	GCUACAGUAACCCUAAGAUTT	AUCUUAGGGUUACUGUAGCTT
c-Fos	GCUACAGUAACCCUAAGAUTT	AUCUUAGGGUUACUGUAGCTT
JunB	CACAAGAUGAACCACGUGATT	UCACGUGGUUCAUCUUGUGTT
JunD	GCCUGGAGGAGAAAGUCAATT	UUGACUUUCUCCUCCAGGCTT
Sox9	AACGAGAGCGAGAAGAGACTT	GGGUCUCUUCUCGCUCUCGTT

### Statistical Analysis

All statistical analyses were carried out using SPSS 16 software. Data, obtained from experiments in duplicate or triplicate and repeated at least three times, are represented as mean ± SD. The unpaired Student’s t test was used to compare two groups with Shapiro-Wilk test for normality test. One-way ANOVA was performed with Levene’s test for homogeneity of variance, followed by the Bonferroni *post hoc* test based on the comparison to be made and the statistical indication of each test. Mauchly’s sphericity test was used for sphericity test. For non-parametric data, difference between groups were evaluated with non-parametric Mann-Whitney U-test, and categorical and binary variables were tested by the Fisher exact test. Statistical significance was accepted at p < 0.05.

## Results

### Stanozolol Prevented Growth Deceleration and Promoted Growth Plate Development in Rats Undergoing GnRHa Treatment

**Figure 1:**
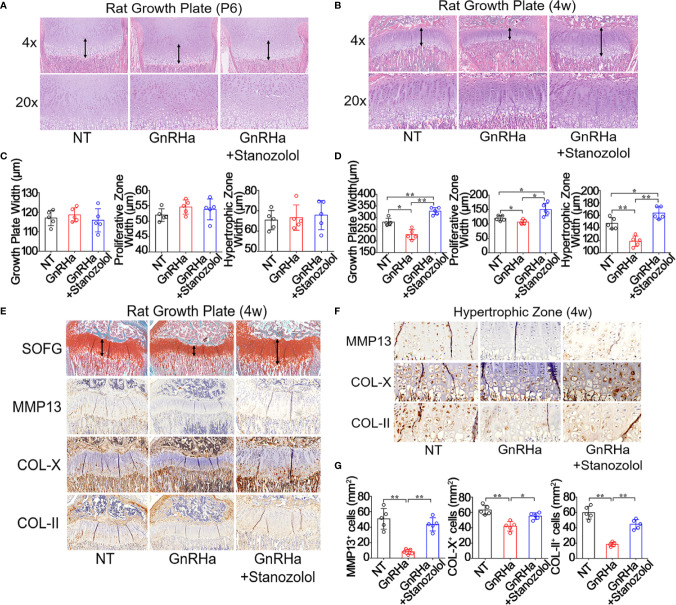
Stanozolol prevented growth deceleration and promoted growth plate development in rats undergoing GnRHa treatment. **(A, B)** H&E staining of the growth plate in rats. **(C, D)** Quantitative analysis of growth plate width in rats. **(E)** SOFG and immunochemical staining analysis of the growth plate in rats. **(F)** Immunochemical staining analysis of the hypertrophic zone of the growth plate in rats. **(G)** Quantitative analysis of positive cell number in hypertrophic zones. Data shown as mean ± SD. n = 5. *p < 0.05 compared between groups; **p < 0.01 compared between groups.

To evaluate the effect of ST on growth plate development, rats were intramuscularly injected with GnRHa (2.5mg/kg) and ST (10mg/kg). H&E staining revealed that there was no significant difference in growth plate width, proliferative zone width, or hypertrophic zone width at postnatal day 6 (**Figures 1A–C**). At postnatal week 4, growth plate width, proliferative zone width, and hypertrophic zone width were significantly decreased in rats given the single GnRHa treatment (Growth plate width: from 281.14 ± 15.63 to 227.12 ± 21.46; Proliferative zone width: from 119.71 ± 8.96 to 106.51 ± 7.74; Hypertrophic zone width: from 147.29 ± 9.24 to 118.57 ± 8.28). However, these same three measurements were significantly increased in the rats receiving the GnRHa/ST combined treatment (Growth plate width: from 227.12 ± 21.46 to 327.53 ± 15.25; Proliferative zone width: from 106.51 ± 7.74 to 149.85 ± 21.78; Hypertrophic zone width: from 118.57 ± 8.28 to 163.83 ± 9.89) (**Figures 1B–D**). SOFG staining showed a similar result for growth plate width (**Figure 1E**). Immunostaining analyses revealed that expressions of MMP13, COL-X, and COL-II were significantly decreased in growth plates with the GnRHa treatment (MMP13: from 51.00 ± 13.36 to 8.20 ± 3.35; COL-X: from 63.20 ± 6.18 to 42.00 ± 6.20; COL-II: from 59.80 ± 8.32 to 18.40 ± 2.70) and increased with the GnRHa/ST combined treatment (MMP13: from 8.20 ± 3.35 to 43.80 ± 8.56; COL-X: from 42.00 ± 6.20 to 55.20 ± 4.32; COL-II: from 18.40 ± 2.70 to 44.60 ± 6.19) (**Figures 1E–G**). These results suggested that ST efficiently prevented growth deceleration and promoted growth plate development in rats undergoing GnRHa treatment.

### Stanozolol Suppressed the Inhibitory Effect of GnRHa to Promote Chondrogenic Differentiation

**Figure 2:**
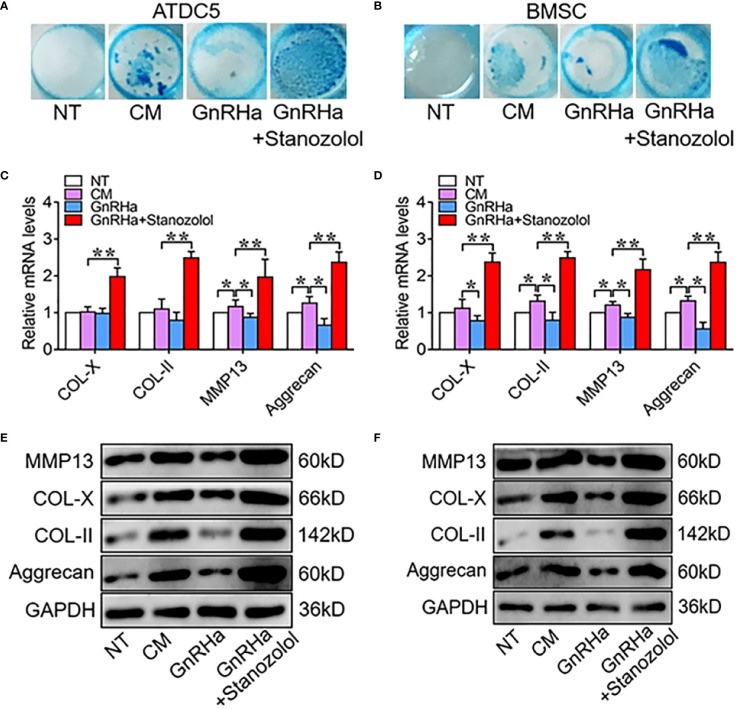
Stanozolol suppressed the inhibitory effects of GnRHa to promote chondrogenic differentiation. **(A, B)** Alcian blue staining of ATDC5 cells and hBMSCs for 9 d. **(C, D)** RT-qPCR analysis of chondrogenic markers in ATDC5 cells and hBMSCs. **(E, F)** Western blot analysis of chondrogenic markers in ATDC5 cells and BMSC. The data are presented as means ± SD from one representative experiment of three independent experiments performed in triplicate. *p < 0.05 compared between groups; **p < 0.01 compared between groups.

To determine the chondro-inductive capacity of ST under GnRHa treatment, ATDC5 and hBMSC cells were treated with GnRHa (5nM) and ST (10nM). The results of alcian blue staining showed that GnRHa significantly suppressed chondrogenic differentiation of ATDC5 and hBMSC cells, and this inhibitory effect was rescued by ST (**Figures 2A, B**). Similar effects were observed in the expressions of chondrogenic relative markers including COL-X, COL-II, MMP13, and Aggrecan at mRNA and protein levels. With GnRHa/ST combined treatment, mRNA levels of COL-X, COL-II, MMP13, and Aggrecan were significantly increased to 1.98-fold, 2.49-fold, 1.97-fold and 2.37-fold in ATDC5 cells; mRNA levels of COL-X, COL-II, MMP13, and Aggrecan were significantly increased to 2.38-fold, 2.48-fold, 2.17-fold and 2.38-fold in hBMSC cells (**Figures 2C–F**). These results suggested that ST suppressed the inhibitory effects of GnRHa to promote chondrogenic differentiation.

### Stanozolol Induced Chondrogenic Differentiation in a Sox9 Dependent Manner

**Figure 3:**
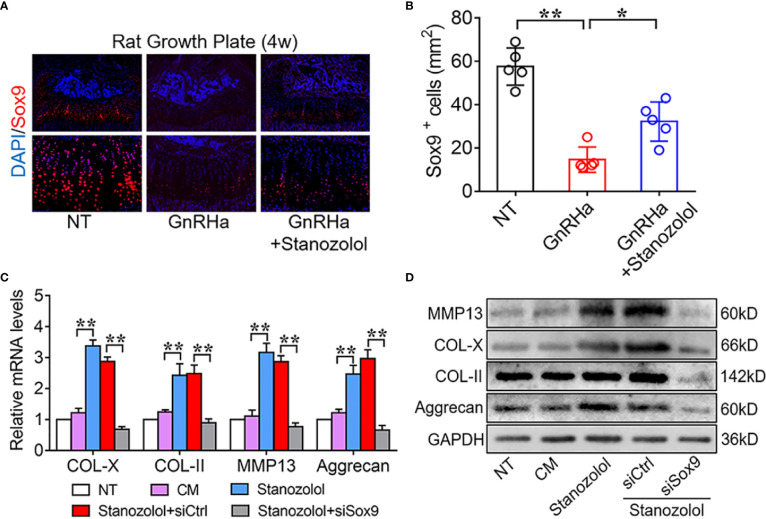
Stanozolol-induced chondrogenic differentiation in a Sox9 dependent manner. **(A)** Immunofluorescence analysis of growth plates in rats. **(B)** Quantitative analysis of Sox9-positive cell number in the growth plates of rats. Data shown as mean ± SD. n = 5. **(C)** RT-qPCR analysis of chondrogenic markers in ATDC5 cells. **(D)** Western blot analysis of chondrogenic markers in ATDC5 cells. The data are presented as means ± SD from one representative experiment of three independent experiments performed in triplicate. *p < 0.05 compared between groups; **p < 0.01 compared between groups.

Since Sox9 is a well-known transcription factor that regulates matrix gene expression in chondrocytes (23, 24), we further explored the potential role of Sox9 in ST promotion of chondrogenic differentiation. The results of immunofluorescence staining revealed that Sox9 positive cells were significantly decreased from 57.60 ± 8.56 to 14.60 ± 5.86 at the growth plates of rats receiving the single GnRHa treatment and increased from 14.60 ± 5.86 to 32.2 ± 9.01 with the GnRHa/ST combined treatment (**Figures 3A, B**). Furthermore, Sox9-specific small interfering RNA (siRNA) was constructed and transfected in ATDC5 cells. The results of PCR and WB showed that Sox9 specific siRNA significantly decreased ST-induced up-regulation of chondrogenic marker expressions at both mRNA and protein levels. With silencing of Sox9, mRNA levels of COL-X, COL-II, MMP13, and Aggrecan were significantly decreased from 2.88 to 0.68-fold, 2.49 to 0.89-fold, 2.87 to 0.77-fold and 2.97 to 0.66-fold in ATDC5 cells (**Figures 3C, D**). These results suggested that ST promoted growth plate development and chondrogenic differentiation in a Sox9 dependent manner.

### Stanozolol Up-Regulated Sox9 Through the JNK/c-Jun Pathway

**Figure 4:**
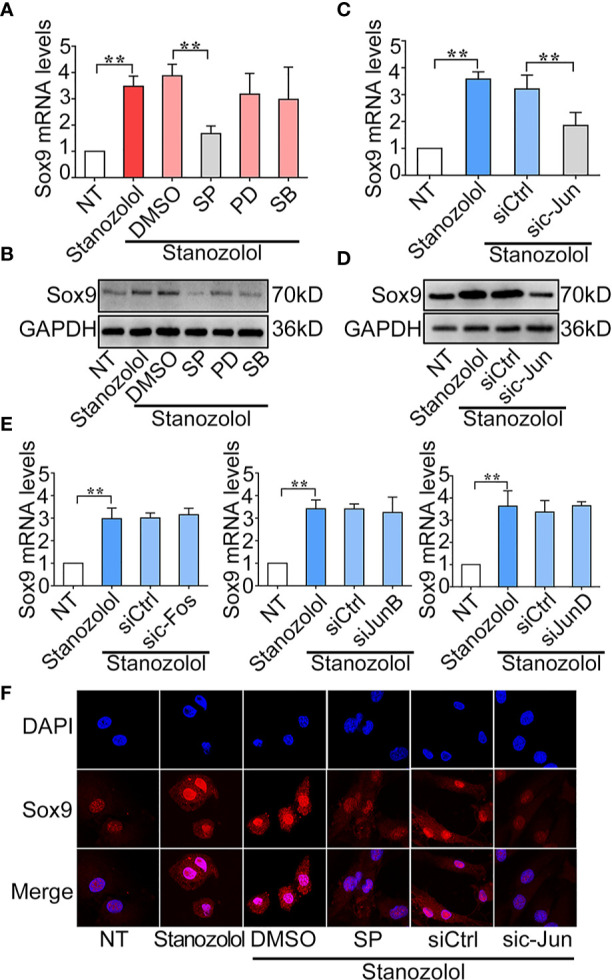
Stanozolol up-regulated Sox9 through the JNK/c-Jun pathway. **(A, B)** RT-qPCR and western blot analysis of Sox9 expression in ATDC5 cells treated with MAPK inhibitors and ST. **(C, D)** RT-qPCR and western blot analysis of Sox9 expression in ATDC5 cells transfected with c-Jun siRNA and treated with ST. **(E)** RT-qPCR analysis of Sox9 expression in ATDC5 cells transfected with other AP-1 subunit siRNA and treated with ST. **(F)** Immunofluorescence analysis of Sox9 expression in ATDC5 cells. Data are presented as means ± SD from one representative experiment of three independent experiments performed in triplicate. **p < 0.01 compared between groups.

To determine the regulatory mechanism of ST-induced Sox9 expression, ATDC5 cells were treated with pharmaceutical inhibitors of MAPKs then stimulated with ST for 24 h. Sox9 expression was then detected with RT-qPCR and western blot. PCR results showed that inhibition of JNK (SP) significantly decreased Sox9 expression from 3.71 to 1.53-fold at both mRNA. Western blot results showed similar result of the expression of Sox9 in ATDC5 cells. (**Figures 4A, B**). In addition, c-Jun was an important transcription factor in the MAPK/JNK pathway. The c-Jun-specific siRNA was constructed and transfected in ADTC5 cells. The PCR results showed that c-Jun specific siRNA significantly decreased ST-induced up-regulation of Sox9 expression from 3.21 to 1.85-fold at mRNA level. Western blot results showed similar result of the expression of Sox9 in ATDC5 cells. (**Figures 4C, D**). In addition, silencing of the expression of the other AP-1 subunits: c-Fos, JunB, and JunD had no effect on ST-induced Sox9 expression (**Figure 4E**). These results indicated that c-Jun, but not c-Fos, JunB, or JunD, played an important role in ST-mediated regulation of Sox9 expression. Immunofluorescence results revealed that the JNK/c-Jun signaling pathway was required for the up-regulation and translocation of Sox9 (**Figure 4F**). These results suggested that ST up-regulated Sox9 through the JNK/c-Jun pathway.

### Stanozolol Promoted Chondrogenic Differentiation and Growth Plate Development Through the JNK/Sox9 Signaling Pathway *In Vivo*

**Figure 5:**
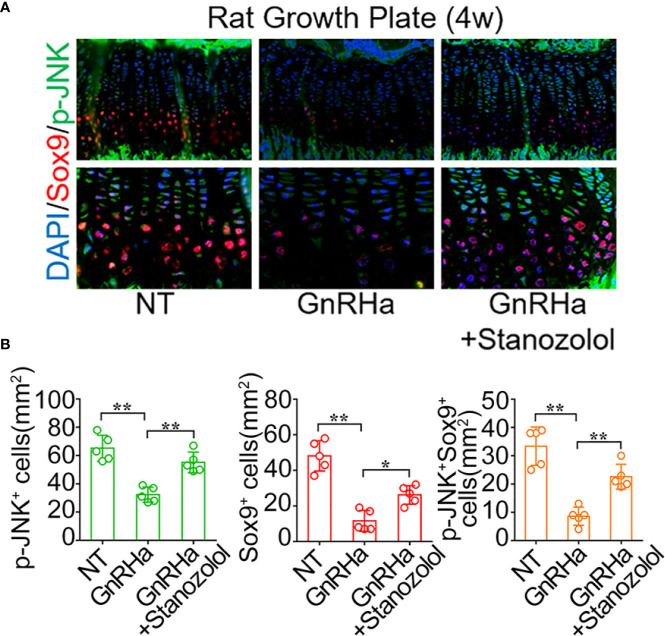
Stanozolol promotes chondrogenic differentiation and growth plate development through the JNK/Sox9 signaling pathway *in vivo*. **(A)** Immunofluorescence analysis of growth plates in rats undergoing GnRHa and ST treatments. **(B)** Quantitative analysis of p-JNK^+^, Sox9^+^, and p-JNK^+^ Sox9^+^ cell number in the growth plates of rats. Data shown as mean ± SD. n = 5. *p < 0.05 compared between groups; **p < 0.01 compared between groups.

To confirm the activation of the JNK/Sox9 signaling pathway in rats undergoing GnRHa/ST combined treatment, we observed the expression of JNK and Sox9 in the growth plate of rats being administered GnRHa and ST treatment. Immunofluorescence staining demonstrated that positive staining of p-JNK and Sox9 was notably decreased at the growth plate after the single GnRHa treatment (p-JNK^+^ cells: from 65.20 ± 9.20 to 32.40 ± 5.37; Sox9^+^ cells: from 48.20 ± 8.58 to 11.60 ± 5.90; p-JNK^+^ Sox9^+^ cells: from 33.40 ± 6.80 to 8.60 ± 3.21), whereas the GnRHa/ST combined treatment led to a significant increase in positive staining of p-JNK and Sox9 at the same region (p-JNK^+^ cells: from 32.40 ± 5.37 to 55.20 ± 7.29; Sox9^+^ cells: from 11.60 ± 5.90 to 26.20 ± 5.26; p-JNK^+^ Sox9^+^ cells: from 8.60 ± 3.21 to 22.60 ± 4.39) (**Figures 5A, B**). These results indicated that ST promoted chondrogenic differentiation and growth plate development through the JNK/Sox9 signaling pathway *in vivo*.

## Discussion

GnRHa is the standard agent for the treatment of CPP with progressive puberty and accelerative growth (1, 2, 25). The efficacy and safety of GnRHa treatment for CPP have been well described (1). However, the side effects of GnRHa such as growth deceleration or the prevention of growth plate development, which lead to a reduction in height velocity, are also reported (3, 9, 11). Thus, an agent which can diminish the side effects of GnRHa and maintain normal growth plate development would theoretically be of great benefit for the treatment of CPP. Previous studies have reported the positive effects of GH administration on height in patients with CPP and showed that the combined use of GH and GnRHa is effective in preventing a decline in growth rate (26–28). However, GH is an expensive medicine and difficult to use in conventional treatment.

ST has a high anabolic to androgenic ratio and has been used to stimulate bone growth in patients with delayed growth and puberty (29). Previous studies reveal that it stimulates a height velocity increase without bone age progression in patients with Turner syndrome undergoing oxandrolone treatment (19). Our previous study also showed that ST promotes the proliferation of growth plate chondrocytes (15). Therefore, we speculated that ST stimulates growth plate development through the promotion of chondrogenic differentiation in patients who were experiencing an inhibitory effect of GnRHa. If that is the case, ST could be a potential therapeutic agent for CPP patients undergoing GnRHa treatment.

In the current study, we made several critical observations that provide insights into the ST-induced chondrogenic differentiation and promotion of growth plate development. Firstly, we found that the prevention of growth plate development and growth deceleration were found in rats given a single GnRHa treatment and that ST abolished the side effects of GnRHa on growth plate development (**Figure 1**). Our previous studies report that ST activates ERα through the MAPK/ERK signaling pathway to promote the proliferation of growth plate chondrocytes (15). Several previous studies of ST have mainly focused on cartilage regeneration in osteoarthritis (OA). Castro et al. report that ST has chondroprotective effects in OA (28). In the latter study, ST treatment reduced the expression of several pro-inflammatory cytokines (MMP1, IL-6, and COX-2) in both normal and inflammatory chondrocytes to prevent the degeneration of cartilage. Spadari et al. also report that ST intra-articular treatment reduces osteophyte formation and subchondral bone reaction and promotes articular cartilage regeneration in OA (30). An important and interesting finding of our *in vitro* experiment that has not been described previously was that ST promoted chondrogenic differentiation and even suppressed the inhibitory effects of GnRHa to promote chondrogenic differentiation (**Figure 2**).

Further, we found that the chondrogenic effect of ST in chondrocytes took place through activation of MAPK/JNK and up-regulation of the expression of Sox9. ST has been reported to induce the activation of MAPK/ERK in other systems (15). In chondrogenic differentiation, the downstream signaling of ST is still unclear. In the current study, we showed that ST promoted chondrogenic differentiation in ATDC5 cells through up-regulating Sox9 gene expression (**Figure 3**). Furthermore, we confirmed that JNK regulated Sox9 expression through regulating the transcriptional factor c-Jun (AP-1 subunit). To our knowledge, this is the first reported description of a ST-JNK/c-Jun-Sox9 signaling cascade in chondrocytes (**Figure 4**). Sox9 is known to be the critical transcriptional factor in growth plate development and chondrogenic differentiation (31, 32).

In our rat model, we found that ST rescued the inhibitory effects of GnRHa to promote growth plate development (**Figure 1**). With the single GnRHa treatment, the activation of JNK was suppressed and the expression of Sox9 was decreased in growth plate chondrocytes. However, with the GnRHa/ST combined treatment, activation of the JNK/c-Jun-Sox9 signaling cascade was rescued in growth plate chondrocytes. This is the first evidence that the JNK/Sox9 signaling cascade is critical for ST-induced growth plate development (**Figure 5**).

There are several limitations to the current study. First, the mechanisms involved in preventing growth plate development with GnRHa and suppressing the inhibitory effect of GnRHa through ST are still unclear. Second, other pathways such Smad1/4 also are critical to chondrogenic differentiation, whether these pathways are involved in chondrogenic effect of ST is needed to further confirm. Third, an animal model established under the background of chondrocyte-specific ablation of ST relative downstream molecules (JNK, c-Jun, Sox9 etc.) would be the best method to specifically verify the effect of ST on growth plate development. Therefore, transgenic animal models should be established to provide more convincing evidence. In addition, clinical data from patients with CPP is also important.

## Conclusions

We demonstrated that ST has a significant chondro-inductive effect and promotes growth plate development through its impact on the JNK/c-Jun/Sox9 signaling pathway. In addition, ST is a potential agent for use in CPP patients treated with GnRHa. Our novel findings may shed light on the mechanism of ST promotion of growth plate development and assist with its clinical application.

## Data Availability Statement

The raw data supporting the conclusions of this article will be made available by the authors, without undue reservation.

## Ethics Statement

The animal study was reviewed and approved by the Medical Ethics Committee of the First Affiliated Hospital of Sun Yat-sen University.

## Author Contributions

SZ and LL contributed equally to this work. Study design: SZ and ZY. Conduction of the study: LL and YT. Data collection: LL, YT, YH, and YL. Data analysis: SZ, LL, and ZY. Data interpretation: YH, YL, and ZY. Drafted the manuscript: SZ and LL. Revised the manuscript content: SZ and LL. Approved the final version of the manuscript: SZ and ZY. All authors take responsibility for the integrity of the data analysis. All authors contributed to the article and approved the submitted version.

## Funding

This work was supported by Guangdong Natural Science Foundation, China [Grant no. 2019A1515010241].

## Conflict of Interest

The authors declare that the research was conducted in the absence of any commercial or financial relationships that could be construed as a potential conflict of interest.

## Abbreviations

GnRHa, Gonadotropin-releasing hormone agonist; CPP, Central Precocious Puberty; ST, stanozolol; GH, Growth Hormone; COL-X, Collagen-X; COL-II, Collagen II; MMP13, Matrix Metalloproteinase 13; SP, JNK specific inhibitor; PD, ERK specific inhibitor; SB, p38 specific inhibitor.

## References

[1] Brito VN Spinola-Castro AM Kochi C Kopacek C Silva PC Guerra-Junior G. Central Precocious Puberty: Revisiting the Diagnosis and Therapeutic Management. Arch Endocrinol Metab (2016) 60(2):163–72. 10.1590/2359-3997000000144 27191050

[2] Chen M Eugster EA. Central Precocious Puberty: Update on Diagnosis and Treatment. Paediatr Drugs (2015) 17(4):273–81. 10.1007/s40272-015-0130-8 PMC5870137 25911294

[3] Carel JC Roger M Ispas S Tondu F Lahlou N Blumberg J. Final Height After Long-Term Treatment With Triptorelin Slow Release for Central Precocious Puberty: Importance of Statural Growth After Interruption of Treatment. French Study Group of Decapeptyl in Precocious Puberty. J Clin Endocrinol Metab (1999) 84(6):1973–8. 10.1210/jcem.84.6.5647 10372696

[4] Pasquino AM Pucarelli I Accardo F Demiraj V Segni M Di Nardo R. Long-Term Observation of 87 Girls With Idiopathic Central Precocious Puberty Treated With Gonadotropin-Releasing Hormone Analogs: Impact on Adult Height, Body Mass Index, Bone Mineral Content, and Reproductive Function. J Clin Endocrinol Metab (2008) 93(1):190–5. 10.1210/jc.2007-1216 17940112

[5] Lazar L Padoa A Phillip M. Growth Pattern and Final Height After Cessation of Gonadotropin-Suppressive Therapy in Girls With Central Sexual Precocity. J Clin Endocrinol Metab (2007) 92(9):3483–9. 10.1210/jc.2007-0321 17579199

[6] Fuqua JS. Treatment and Outcomes of Precocious Puberty: An Update. J Clin Endocrinol Metab (2013) 98(6):2198–207. 10.1210/jc.2013-1024 23515450

[7] Vatopoulou A Roos E Daniilidis A Dinas K. Long-Term Effects of Treatment of Central Precocious Puberty With Gonadotropin-Releasing Hormone Analogs Every Three Months. Gynecol Endocrinol (2020) 36(12):1–3. 10.1080/09513590.2020.1770723 32484003

[8] Vuralli D Ozon ZA Gonc EN Alikasifoglu A Kandemir N. Long-Term Effects of GnRH Agonist Treatment on Body Mass Index in Girls With Idiopathic Central Precocious Puberty. J Pediatr Endocrinol Metab (2020) 33(1):99–105. 10.1515/jpem-2019-0214 31804960

[9] Kauli R Galatzer A Kornreich L Lazar L Pertzelan A Laron Z. Final Height of Girls With Central Precocious Puberty, Untreated Versus Treated With Cyproterone Acetate or GnRH Analogue. A Comparative Study With Re-Evaluation of Predictions by the Bayley-Pinneau Method. Horm Res (1997) 47(2):54–61. 10.1159/000185432 9030968

[10] Massart F Federico G Harrell JC Saggese G. Growth Outcome During GnRH Agonist Treatments for Slowly Progressive Central Precocious Puberty. Neuroendocrinology (2009) 90(3):307–14. 10.1159/000231994 19641297

[11] Glab E Wikiera B Bieniasz J Barg E. The Influence of GnRH Analog Therapy on Growth in Central Precocious Puberty. Adv Clin Exp Med (2016) 25(1):27–32. 10.17219/acem/31433 26935495

[12] Pasquino AM Municchi G Pucarelli I Segni M Mancini MA Troiani S. Combined Treatment With Gonadotropin-Releasing Hormone Analog and Growth Hormone in Central Precocious Puberty. J Clin Endocrinol Metab (1996) 81(3):948–51. 10.1210/jcem.81.3.8772556 8772556

[13] Pucarelli I Segni M Ortore M Arcadi E Pasquino AM. Effects of Combined Gonadotropin-Releasing Hormone Agonist and Growth Hormone Therapy on Adult Height in Precocious Puberty: A Further Contribution. J Pediatr Endocrinol Metab (2003) 16(7):1005–10. 10.1515/jpem.2003.16.7.1005 14513877

[14] Pucarelli I Segni M Ortore M Moretti A Iannaccone R Pasquino AM. Combined Therapy With GnRH Analog Plus Growth Hormone in Central Precocious Puberty. J Pediatr Endocrinol Metab (2000) 13(Suppl 1):811–20. 10.1515/jpem.2000.13.s1.811 10969926

[15] Zhu SY Li YH Ma HM Huang TT Luo HB Dou J. Stanozolol Regulates Proliferation of Growth Plate Chondrocytes Via Activation of ERalpha in GnRHa-treated Adolescent Rats. J Pediatr Endocrinol Metab (2011) 24(5-6):275–81. 10.1515/jpem.2011.183 21823523

[16] Lampit M Golander A Guttmann H Hochberg Z. Estrogen Mini-Dose Replacement During GnRH Agonist Therapy in Central Precocious Puberty: A Pilot Study. J Clin Endocrinol Metab (2002) 87(2):687–90. 10.1210/jcem.87.2.8242 11836305

[17] Xiong H Chen HS Du ML Li YH Ma HM Su Z. Therapeutic Effects of Growth Hormone Combined With Low-Dose Stanozolol on Growth Velocity and Final Height of Girls With Turner Syndrome. Clin Endocrinol (Oxf) (2015) 83(2):223–8. 10.1111/cen.12785 25824243

[18] Vergallo C Torrieri G Provenzani R Miettinen S Moslova K Varjosalo M. Design, Synthesis and Characterization of a PEGylated Stanozolol for Potential Therapeutic Applications. Int J Pharm (2020) 573:118826. 10.1016/j.ijpharm.2019.118826 31715352

[19] Gawlik A Gawlik T Koehler B Malecka-Tendera E Augustyn M Woska W. Influence of Hormonal Therapy on Growth Rate and Bone Age Progression in Patients With Turner Syndrome. Endokrynol Pol (2005) 56(2):136–44. 16335681

[20] Li X Wang J Zhan Z Li S Zheng Z Wang T. Inflammation Intensity-Dependent Expression of Osteoinductive Wnt Proteins is Critical for Ectopic New Bone Formation in Ankylosing Spondylitis. Arthritis Rheumatol (2018) 70(7):1056–70. 10.1002/art.40468 29481736

[21] Siebler T Robson H Shalet SM Williams GR. Dexamethasone Inhibits and Thyroid Hormone Promotes Differentiation of Mouse Chondrogenic ATDC5 Cells. Bone (2002) 31(4):457–64. 10.1016/s8756-3282(02)00855-4 12398940

[22] Li X Li Z Wang J Li Z Cui H Dai G. Wnt4 Signaling Mediates Protective Effects of Melatonin on New Bone Formation in an Inflammatory Environment. FASEB J (2019) 33(9):10126–39. 10.1096/fj.201900093RR 31216173

[23] Barter MJ Gomez R Hyatt S Cheung K Skelton AJ Xu Y. The Long non-Coding RNA ROCR Contributes to SOX9 Expression and Chondrogenic Differentiation of Human Mesenchymal Stem Cells. Development (2017) 144(24):4510–21. 10.1242/dev.152504 PMC5769619 29084806

[24] Liu CF Samsa WE Zhou G Lefebvre V. Transcriptional Control of Chondrocyte Specification and Differentiation. Semin Cell Dev Biol (2017) 62:34–49. 10.1016/j.semcdb.2016.10.004 27771362 PMC5318237

[25] Ramos CO Latronico AC Cukier P Macedo DB Bessa DS Cunha-Silva M. Long-Term Outcomes of Patients With Central Precocious Puberty Due to Hypothalamic Hamartoma After GnRHa Treatment: Anthropometric, Metabolic, and Reproductive Aspects. Neuroendocrinology (2018) 106(3):203–10. 10.1159/000477584 28558376

[26] Antoniazzi F Zamboni G Bertoldo F Lauriola S Tato L. Bone Development During GH and GnRH Analog Treatment. Eur J Endocrinol (2004) 151(Suppl 1):S47–54. 10.1530/eje.0.151s047 15339244

[27] Jung MK Song KC Kwon AR Chae HW Kim DH Kim HS. Adult Height in Girls With Central Precocious Puberty Treated With Gonadotropin-Releasing Hormone Agonist With or Without Growth Hormone. Ann Pediatr Endocrinol Metab (2014) 19(4):214–9. 10.6065/apem.2014.19.4.214 PMC4316408 25654068

[28] Kim MS Koh HJ Lee GY Kang DH Kim SY. Comparing Adult Height Gain and Menarcheal Age Between Girls With Central Precocious Puberty Treated With Gonadotropin-Releasing Hormone Agonist Alone and Those Treated With Combined Growth Hormone Therapy. Ann Pediatr Endocrinol Metab (2019) 24(2):116–23. 10.6065/apem.2019.24.2.116 PMC6603606 31261476

[29] Adachi M Takayanagi R. [Effect of Anabolic Steroids on Osteoporosis]. Clin Calcium (2008) 18(10):1451–9. CliCa081014511459 18830042

[30] Spadari A Romagnoli N Predieri PG Borghetti P Cantoni AM Corradi A. Effects of Intraarticular Treatment With Stanozolol on Synovial Membrane and Cartilage in an Ovine Model of Osteoarthritis. Res Vet Sci (2013) 94(3):379–87. 10.1016/j.rvsc.2012.11.020 23352201

[31] Kozhemyakina E Lassar AB Zelzer E. A Pathway to Bone: Signaling Molecules and Transcription Factors Involved in Chondrocyte Development and Maturation. Development (2015) 142(5):817–31. 10.1242/dev.105536 PMC4352987 25715393

[32] Dy P Wang W Bhattaram P Wang Q Wang L Ballock RT. Sox9 Directs Hypertrophic Maturation and Blocks Osteoblast Differentiation of Growth Plate Chondrocytes. Dev Cell (2012) 22(3):597–609. 10.1016/j.devcel.2011.12.024 22421045 PMC3306603

